# Evaluation of alcohol intoxication on primary and secondary haemostasis – results from comprehensive coagulation testing

**DOI:** 10.1007/s00414-025-03449-7

**Published:** 2025-02-22

**Authors:** Ronja Sabine Schmölders, Till Hoffmann, Derik Hermsen, Michael Bernhard, Fritz Boege, Michael Lau, Benno Hartung

**Affiliations:** 1 Institute of Legal Medicine, University Hospital Düsseldorf, Heinrich-Heine University, Moorenstrasse 5, 40225 Düsseldorf, Germany; 2 Department of Psychiatry and Psychotherapy, Medical Faculty, University Hospital Düsseldorf, Heinrich-Heine University, Bergische Landstrasse 2, 40629 Düsseldorf, Germany; 3 Institute of Transplantation Diagnostics and Cell Therapeutics, University Hospital Düsseldorf, Heinrich-Heine University, Moorenstrasse 5, 40225 Düsseldorf, Germany; 4 Central Institute of Clinical Chemistry and Laboratory Diagnostics, University Hospital Düsseldorf, Heinrich-Heine University, Moorenstrasse 5, 40225 Düsseldorf, Germany; 5 Emergency Department, University Hospital Düsseldorf, Heinrich-Heine University, Moorenstrasse 5, 40225 Düsseldorf, Germany; 6 Mathematical Institute, Heinrich Heine University, Universitaetsstr. 1, 40225 Düsseldorf, Germany; 7 Institute of Legal Medicine, University Hospital Essen, University Duisburg-Essen, Hufelandstraße 55, 45147 Essen, Germany

**Keywords:** Thromelastography, Impedance aggregometry, Platelet function, Alcoholisation, Post mortem, Blood coagulation

## Abstract

**Background:**

Alcohol intoxication is known to affect blood coagulation. The specific effects remain poorly understood. Here, we investigate the impact of severe alcohol intoxication on blood clotting by comprehensive coagulation testing.

**Methods:**

A prospective study included 21 patients admitted to the emergency department of University Hospital Düsseldorf with severe alcohol intoxication (target blood alcohol concentration > 2 g/l). Platelet function and coagulation was compared between states of alcohol intoxication and soberness using multiple platelet function analysis, thrombelastography and determination of single coagulation factors. The same test panel was used to study in vitro-effects of ethanol on coagulation.

**Results:**

Blood alcohol was correlated with impaired platelet aggregation determined in vivo by functional testing employing ADP and ASPI stimulation. Blood alcohol-associated coagulation impairment was not detectable by thrombelastography or clotting factor measurements. Blood alcohol was negatively correlated with von Willebrand factor ratio and clot strength. The association of elevated blood alcohol with impaired coagulation could not be replicated in vitro.

**Discussion:**

Our findings suggest that alcohol impairs primary hemostasis by reducing platelet function, while secondary hemostasis remains largely unaffected. Reversion of effects upon sobering suggest a rather direct impact of alcohol on platelet function. That effect was, however, not replicated in vitro possibly implicating involvement of vascular factors.

**Conclusion:**

Blood alcohol has a potentially negative impact on platelet function, which should be considered in the clinical management of intoxicated patients, especially in emergency settings. Potential bleeding risks due to increased blood alcohol are possibly detected by analysis of platelet function, while not by thrombelastography or plasmatic coagulation tests.

**Supplementary Information:**

The online version contains supplementary material available at 10.1007/s00414-025-03449-7.

## Introduction

Haemostasis is a vital multi-stage process that ultimately leads to the formation of a fibrin complex to handle vascular injury [1]. At the beginning of primary haemostasis, vascular haemostasis occurs and a white thrombus is formed via the three stages of platelet adhesion, activation, and aggregation. During these processes, various proteins and messenger substances such as ADP and von Willebrand factor are released. Afterwards, plasmatic coagulation leads to a stable fibrin network via various steps. In the individual steps, plasma proteins (coagulation factors) are activated by proteolytic cleavage. The substances involved in the entire coagulation process can be measured individually. In addition, the times or effectiveness of the processes can be determined.

The observation that blood of alcohol intoxicated persons fails to coagulate post mortem can already be found in historic publications [2]. Various mechanisms have been proposed by which ethanol could influence blood coagulation:

Thrombelastometric analyses in vitro show an inhibition of blood coagulation at increasing blood alcohol concentrations (BAC). Fibrinolytic activity determined in vitro was found to be significantly impaired above a BAC of 1 g/kg, which suggests a prothrombotic effect of ethanol [3]. Inhibition of fibrinolysis at high ethanol levels was also confirmed by in vivo-studies [4, 5] of men (*N* = 50, 26 +—4.6 years) who had consumed various amounts of beer or red wine (20 g, 40 g, 60 g or 80 g ethanol). They exhibited a prolonged (several hours) and significant decrease of fibrinolytic activity, possibly due to concomitant increases in plasminogen-activator-inhibitor antigen (PAI-1) [4].

Confoundingly, in vivo-thrombelastometric analyses carried out in the context of socially acceptable ethanol consumption (*N* = 58, w = m, control group 36.7 and drinkers 29.9 years on average) indicated that male individuals tend to hypocoagulate (decreased rate of fibrin formation, decreased clot strength, and a decreased rate of fibrin cross-linking), while female participants do not [6].

In a more recent ex-vivo study (*N* = 20, age 29.4, m = 11, w = 9), thrombelastometric testing following moderate ethanol consumption (up to max. 1 g/kg) revealed a lower clot strength in the fibrinogen function test (FibTEM), while the extrinsic system test (ExTEM) and fibrinolysis showed no significant changes [7]. A divergent observation was made by a Korean study examining coagulation in patients of a trauma center (*N* = 686, m = 525, median age = 55 y) with and without alcohol intoxication. Alcoholised patients exhibited a prolonged clotting time (CT) and reduced fibrinolysis indicating a predominant impact of ethanol on ExTEM [8].

However, trauma above Injury Severity Score = 4 points, can be associated with hypercoagulation. Trauma-associated hypercoagulation is most pronounced during the first 24 h post trauma, biased towards women, and can only be detected by thrombelastography as opposed to clotting tests such as INR and PTT [9]. A study on the overlay of injury and alcohol intoxication (*N* = 448) confirmed hypocoagulation detectable by thrombelastometric examinations but not by clinical indications such as increased bleeding [10]. Follow-up studies of that cohort demonstrated alcohol-induced impairment of clot formation and inhibition of fibrinolysis [11].

Given the wide-spread and long-standing use of alcohol as stimulant and psychoactive substance [12] alcohol intoxication with or without accompanying injuries is commonly found not only post mortem, but also in emergency departments [13]. The alcohol-induced increase in bleeding risk might thus change medicolegal implications of injury combined with alcohol intoxication, raising the following questions:

(i) How does coagulation change with very high in-vivo BACs (above 1 g/l) with or without accompanying injury?
(ii) Which analyses might be helpful to identify such alcohol-induced coagulation dysfunctions?

To address these questions, we have performed a prospective study approved by the ethics committee of the University Hospital Düsseldorf (study no.: 2019–483).

## Material and methods

### Patients

The study encompasses patients (*N* = 21, m = 14, median age = 51y, range 20–82 years) of the central emergency room of University Hospital Düsseldorf. Patients were included if at least 18 years old and exhibiting clinical signs of severe alcoholisation (clinical assessment as severe alcohol influence with e.g. foetor alcoholicus, slurred speech, significant gait unsteadiness and/or nausea/vomiting; breath alcohol concentration above 1 mg/l; clinical BAC of 2 g/l or above). Preliminary informed consent was obtained in the state of intoxication from the patients themselves or accompanying relatives. Final informed consent was obtained on the next day in the state of soberness. If the patient refused to participate, all results would be deleted and blood specimen would be disposed. Had the patient left hospital without an opportunity to clarify the question of consent, so far obtained results were stored until the patient could be asked for consent. If the patient could not be contacted, data and specimen would be deleted resp. disposed, too. Some patients agreed to the examination of blood that was already taken but refused further blood sampling, leading to incomplete data.

Exclusion criteria were chronic alcohol abuse, suffering from established alcoholic liver injury, treatment with direct oral anticoagulants (DOACS) for up to 24 h prior to blood sampling.

### Experimental set-up

When a person was potentially eligible, study blood sampling (six tubes of citrate blood, three tubes of EDTA blood—3 ml each—and two tubes of serum blood with 7.5 ml each) was carried out together with medically indicated routine sampling. In addition, the injury status was assessed.

Multiple electrode impedance platelet aggregometer (Multiplate®) and thrombelastography (ROTEM®) were carried out within four hours (average one to two hours). The remaining blood specimen were frozen until the question of informed consent was clarified as other tests could be postponed. The freezing procedure was carried out according to the manufacturer’s recommendations, which are based on the CLSI (previous name: NCCLS) guidelines for pre-analytics [14]. All coagulation samples for factor analysis were centrifuged twice and the plasma was removed between centrifugations and transferred to a new tube in order to obtain plasma that was as Tc-free as possible. Subsequently, rapid freezing at −20 °C. Thawing procedure also according to the recommendations of the CLSI guidelines [14]. The measured parameters are not affected by the freezing process. If test persons gave informed consent to be included into the study in the state of soberness, the medical records were taken into account and a both postponed blood analysis and follow up blood collection and analysis were performed.

Blood from the state of alcohol intoxication was analyzed regarding rotational thrombelastometry, impedance aggregometry, (both measured at the Institute of Transplantation Diagnostics and Cell Therapeutics, University Hospital Düsseldorf), BAC, carbohydrate-deficient transferrin (CDT), plasma based global coagulation tests – international normalized ratio (INR)/thromboplastin by Quick, activated partial thromboplastin time (aPTT), thrombin time –, single factor parameters – von Willebrand factor (vWF) antigen (ag)/activity/ratio, coagulation factoractivities II, V, VII, VIII, IX, X, XI, XII, XIII) as well as platelet count and function (these values were all measured at the Central Institute of Clinical Chemistry and Laboratory Diagnostics, University Hospital Düsseldorf).

In the second blood sample (drawn in the state of soberness), parameters for hepatocellular damage, synthesis disorders and cholestasis – Aspartat aminotransferase (AST), Alanine aminotransferase (ALT), glutamate dehydrogenase (GLDH), albumin, γ-glutamyltransferase (*γ*-GT), alkaline phosphatase (AP), direct/indirect and total bilirubin, cholinesterase were determined in addition to the above-mentioned coagulation parameters. C-reactive protein (CRP) and erythrocyte indices (mean corpuscular volume – MCV) from initial testing were taken from the medical records (if available). All these parameters of the second sample were measured at the Central Institute of Clinical Chemistry and Laboratory Diagnostics, University Hospital Düsseldorf using accredited routine diagnostic procedures.

Subsequently, and separately from the tests described above, we carried out a Multiplate® in-vitro test series with blood from one healthy fasting person (female, 27 years). After taking the blood sample, we added different amounts of ethanol in vitro so to reach BAC of 0 g/kg, 1 g/kg, 2 g/kg and 4 g/kg. We then carried out Multiplate® analyses. These analyses were performed after 30 min, 2 h and 3.5 h to assess whether the time interval of alcohol contact to the blood is relevant for the results.

All tests were carried out using standardized procedures.

### Laboratory analyses

#### Platelet function analyses (Multiplate®)

*Multiplate®* is a tool for testing platelet function (Multiplate®, Roche Diagnostics) by using three different activators. Adenosine-diphosphate (ADP) test is sensitive to intrinsic platelet function deficiencies as well as ADP-receptor inhibition by antiplatelet agents. Arachidonic acid (ASPI) test is an assessment of globally suppressed platelet function, and thrombin receptor activating protein-6 (TRAP-6) test allows to assess platelet function independently from antiplatelet drugs other than GPIIb-IIIa antagonists. Detailed information can be found in the manufacturer’s brochure [15].

It should be noted that this method used with whole blood is not suitable for reliably ruling out a platelet dysfunction; it is normally used to monitor therapy with platelet aggregation inhibitors. The method of light transmission aggregometry recommended by the German AWMF guideline [16] was not available 24/7.

#### Thrombelastography (ROTEM®)

ROTEM® analyzer is used to measure functional coagulation. Three different tests (ExTEM, InTEM and FibTEM) were used to assess the different parts of the coagulation system [17].

Increased or decreased values indicate, for example, impaired coagulation activation or abnormal clot formation in the corresponding area of coagulation.

#### Coagulation parameters

Coagulation factors (II, V, VII, VIII, IX, X, XI, XII and XIII), vWF Ag, aPTT, Quick were tested by Turbidimetry (CS 5100 System, Sysmex; reagents from Siemens Healthineers).

#### The following remaining parameters were measured using the methods listed accordingly

Clinical BAC: Photometry, Cobas 8000, Module 701, Roche Diagnostics.

CDT: Immunonephelometry, BN II Nephelometer, Siemens Healthineers.

Platelet count: Impedance measurement, XN systems, from Sysmex.

ALT, AST, yGT: Photometry, Cobas 8000, Module 701, Roche Diagnostics.

## Results

### Test persons

Twenty-one patients were included (14 males, 7 females). Six patients consented to a second blood sampling for study reasons (4 males, 2 females). 15 patients agreed to include samples taken resp. analyzed in advance for study reasons. All other samples resp. results were disposed.

The test persons’ average age was 49.9 years (median 51 years). The mean age of the male test persons was 54.9 years and ranged from 24 to 82 years (median 60.5). The age of the female test persons ranged from 20 to 57 years (median 41) and the mean age was 39.9 years.

Of the 21 test subjects, eleven (7 males, 4 females) had suffered from trauma. Traumas ranged from hematomas or bruises to minor abrasions, cuts or lacerations. No polytrauma patients were included.

The average BAC at admission was 2.49 g/l (trauma 2.52 g/l; non-trauma 2.45 g/l; median 2.69 g/l). BAC ranged from 1.25 g/l to 3.79 g/l.

### Medication taken

It was only possible to determine the medication status of those six people who allowed a second blood sample to be taken in the state of soberness. Three of them did not take any medication. One person only took macrogol on demand. In addition, one person was taking lamotrigine, fluoxetine, mirtazapine, zopiclone, pantoprazole, baclofen, tizanidine, levodopa, gabapentin and the last person was taking ASS, ramipril and adolipin. Results of the last two persons are highlighted in the figures.

### Effects of blood alcohol on platelet function (Multiplate® analyses)

**Fig. 1:**
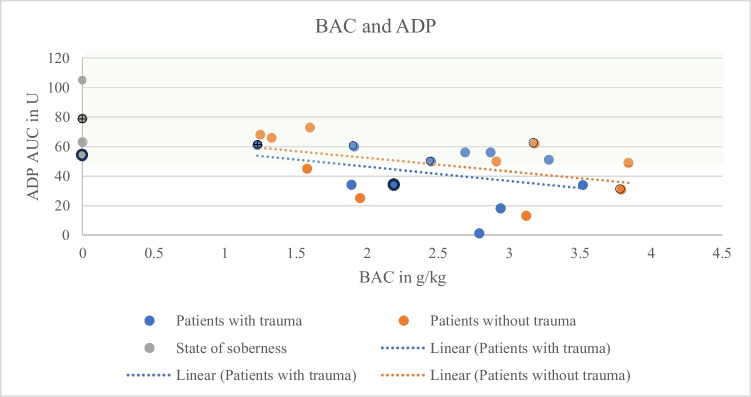
ADP values (y-axis) in relation to the determined forensic BACs (x-axis). Orange: Patients without trauma, correlation coefficient r = −0.46, *p* = 0.18. Blue: Patients with trauma, correlation coefficient r = −0.34, *p* = 0.31. Grey: State of soberness. Hatched green area: Reference area. Dotted: Regression lines. ⨁: patient with fluoxetine and gabapentine.  : patient with ASS, ○: patients without medication

**Fig. 2:**
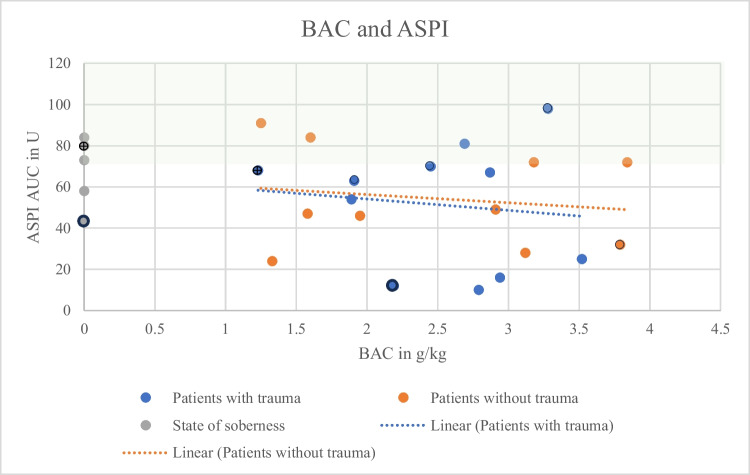
ASPI values (y-axis) in relation to the determined forensic BACs (x-axis). Orange: Patients without trauma, correlation coefficient r = −0.17, *p* = 0.66. Blue: Patients with trauma, correlation coefficient r = −0.12, *p* = 0.72. Grey: State of soberness. Hatched green area: Reference area. Dotted: Regression lines. ⨁: patient with fluoxetine and gabapentine.  : patient with ASS, ○: patients without medication

**Fig. 3:**
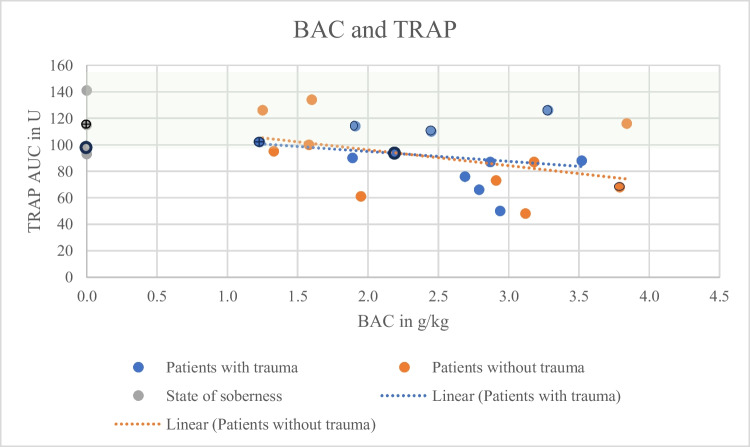
TRAP values (y-axis) in relation to the determined forensic BACs (x-axis). Orange: Patients without trauma, correlation coefficient r = −0.43, *p* = 0.28. Blue: Patients with trauma, correlation coefficient r = −0.23, *p* = 0.50. Grey: State of soberness. Hatched green area: Reference area. Dotted: Regression lines. ⨁: patient with fluoxetine and gabapentine.  : patient with ASS, ○: patient without medication

**Fig. 4:**
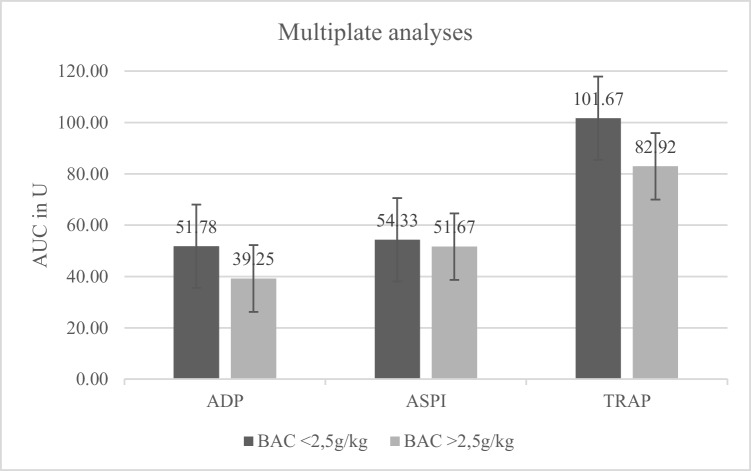
Mean values for BAC < 2.5 g/l (black) compared to BAC > 2.5 g/l (grey) for ADP (reference interval 53U-122U, t-test: *p* = 0.07), ASPI (reference interval 74U-136U, t-test: *p* = 0.41) and TRAP (reference interval 94U-156U, t-test: *p* = 0.04)

Both groups, with (*N* = 11) and without (*N* = 10) trauma, showed a decreasing aggregation capacity with increasing BAC (Figs. 1, 2, and 3). Correlation coefficients and p-values are shown in Table 1. Mean values were lower for all types of stimulation (Fig. 4; ADP and BAC < 2.5 g/l = 44.40% of the values were below the reference area, > 2.5 g/l = 75% of the values were below the reference area, ASPI and BAC < 2.5 g/l = 66.67%, > 2.5 g/l = 83.33%, TRAP and BAC < 2.5 g/l = 33.33%, > 2.5 g/l = 75%) for BACs below 2.5 g/l (*N* = 10) as compared to > 2.5 g/l (*N* = 11). ADP values were regularly below the reference interval (53U-122U; Fig. 1). TRAP values differed significantly between groups below and above 2.5 g/kg (t-test, *p* = 0.04).

**Table 1:** Correlation coefficients and *p*-values in Multiplate® separated by groups (trauma / non-trauma)

Activator	Patient group	Correlation coefficient (r)	*p*-value
ADP	Patients with trauma	0.34	0.31
ADP	Patients without trauma	0.46	0.18
ASPI	Patients with trauma	0.12	0.72
ASPI	Patients without trauma	0.17	0.66
TRAP	Patients with trauma	0.23	0.50
TRAP	Patients without trauma	0.43	0.28

**Fig. 5:**
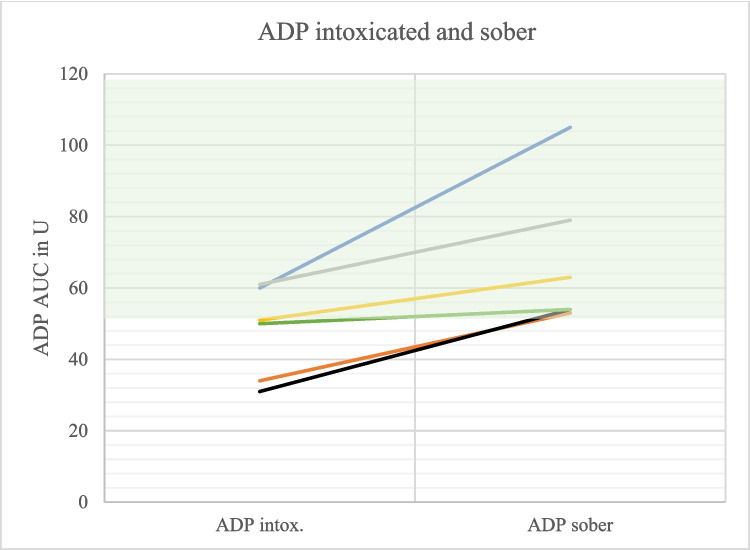
Comparison of ADP values intoxicated and sober (*N* = 6). The different colors represent the different test persons. Hatched green area: Reference area. Intox. = Intoxicated

Multiplate® analyses in the state of soberness (*N* = 6; 1 without trauma, 5 with trauma) showed that the ADP value of an intoxicated person was lower than the sober ADP value of the same person in all cases (Fig. 5). Remarkably, ADP values were within the reference interval in the state of soberness in all cases (Figs. 1 and 5).

**Fig. 6:**
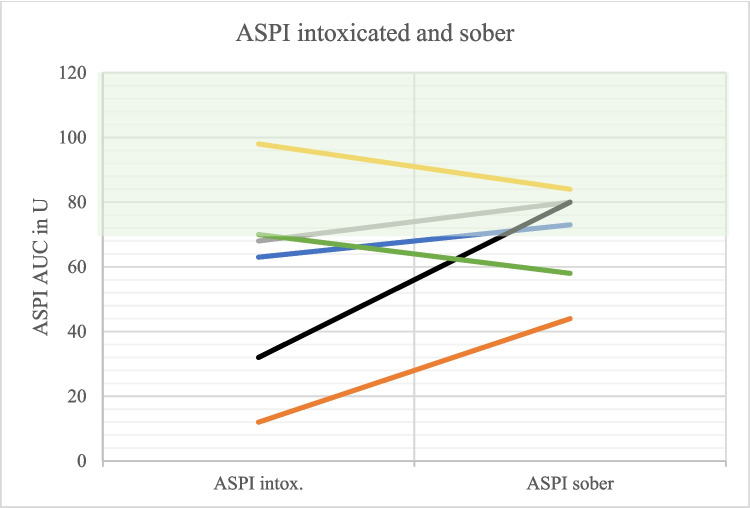
Comparison of ASPI values intoxicated and sober (*N* = 6). The different colors represent the different test persons. Hatched green area: Reference area. Intox. = Intoxicated

**Fig. 7:**
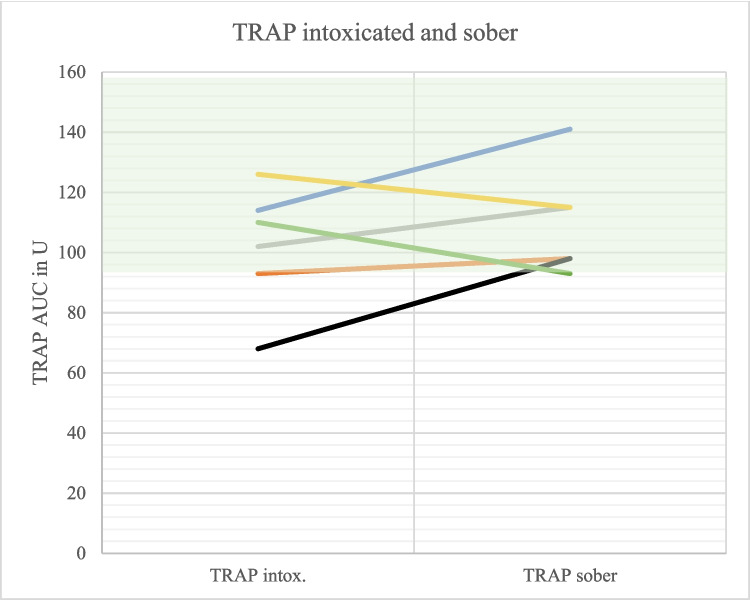
Comparison of TRAP values intoxicated and sober (*N* = 6). The different colors represent the different test persons. Hatched green area: Reference area. Intox. = Intoxicated

Detoxing was associated with increasing aggregometric capacity in 6/6 patients (ADP test), 4/6 pts. (ASPI test) and 4/6 pts. (TRAP test), respectively (Figs. 5, 6, and 7).

### Effects of blood alcohol on platelet function (Multiplate® analyses) in vitro

In-vitro analyses (*N* = 1) did not reveal the observations mentioned above. After 30 min and 2 h, normal results were obtained. Analyses that showed conspicuous Multiplate*®* results (outside the reference area) were all carried out about 3.5 h after adding ethanol:

The ADP test was slightly below the normal range in all three alcohol-treated blood samples (1 g/kg: 44U, 2 g/kg: 46U, 4 g/kg:31U), ASPI test values were decreased at 1 g/kg (71U) and 2 g/kg (62U) and the TRAP test was only decreased in the 2 g/kg specimen (76U).

### Effects of blood alcohol on thrombelastography (ROTEM® analyses)

In the ROTEM® analyses (*N* = 21), a singular patient each in the ExTEM and InTEM analysis showed a prolonged clotting time. Clot firmness in the FibTEM test was increased in four patients. No correlation was observed between thrombelastometric parameters and BAC. All results can be found in Supplement 1.

Results from extrinsic and intrinsic as well as von Willebrand factor testing are shown in Table 2 (measuring the coagulation factors, only possible in 19 test persons):

**Table 2:** Extrinsic, intrinsic and von Willebrand factor test results

Coagulation factors	Normal range (%) Numbers above Numbers below	Mean (%) patients with trauma patients without trauma	Median (%) patients with trauma patients without trauma	Range (%) patients with trauma patients without trauma
Factor II	70 - 100	96.74	90.00	74-146
	4	*93.20*	*88.50*	*74-119*
	0	*100.67*	*92.00*	*84-146*
Factor V	70 - 100	84.95	85.00	31-160
	4	*85.10*	*93.50*	*33-120*
	5	*84.78*	*71.00*	*31-160*
Factor VII	70 - 140	118.47	110.00	76-172
	6	*119.50*	*126.00*	*76-160*
	0	*117.33*	*105.00*	*85-172*
Factor VIII	70 - 200	119.26	106.00	63-232
	2	*121.40*	*104.50*	*76-216*
	1	*116.89*	*106.00*	*63-232*
Factor IX	70 - 110	101.79	96.00	75-165
	5	*100.20*	*99.00*	*82-136*
	0	*103.56*	*96.00*	*75-165*
Factor X	70 - 140	108.79	109.00	74-169
	1	*109.20*	*108.00*	*88-133*
	0	*108.33*	*109.00*	*74-169*
Factor XI	70 - 120	94.95	90.00	65-136
	11	*96.90*	*96.00*	*65-134*
	0	*92.78*	*84.00*	*69-136*
Factor XII	80 - 120	96.63	88.00	59-154
	3	*98.40*	*99.00*	*76-135*
	4	*94.67*	*81.00*	*59-154*
Factor XIII	80 - 120	*128.57*	138.00	85-165
	13	*128.73*	*138.00*	*85-165*
	0	*128.40*	*134.50*	*86-165*
Quick	77 -120	104.67	107.50	84-140
	5	*102.44*	*107.00*	*88-140*
	0	*106.89*	*110.00*	*84-126*
aPPTT*	20-40	24.68	25	21-32
	0	*25.40*	*25*	*22-32*
	0	*23.89*	*24*	*21-27*
vWF-Activity	48 – 240	167.71	164.50	84.6-237.3
	1	*163.42*	*153.00*	*84.6-226*
	0	*172.43*	*170.55*	*101.7-237.3*
vWF-Antigen	50 – 160	167.57	151.70	102.8-245.3
	10	*161.15*	*151.70*	*102.8-237.4*
	0	*174.62*	*160.95*	*119.9-245.3*

*in seconds not in %

Overall, no significant deficits were observed, while increased factor antigen was a frequent finding.

For activities of factors II positive correlations were found with BAC (*N* = 19; Pearson’s correlation coefficient r = 0.20, p = 0.41). The von Willebrand factor ratio (activity / antigen) as an indicator for large multimer function was negatively correlated with BAC (*N* = 21; normal range: > 0.700; mean: 0.999; median: 0.96; range: 0.76–1.27; correlation coefficient r = −0.41; *p* = 0.06.).

### Effects of blood alcohol on chemistry parameters

Clinical chemistry parameters (AST, ALT, GLDH, MCV, albumin, gamma-GT, alkaline phosphatase, direct/indirect and total bilirubin, cholinesterase) showed no abnormalities, with the exception of two subjects (both with trauma, Patient A: male, 61 years, Patient B: female, 34 years), who showed elevated transaminases (ALT: 330 U and 128 U, AST: 183 U and 277 U, gamma-GT: 256 and 916 U).

Patient A presented very low values in the Multiplate® analyses (ADP = 18 U, ASPI = 16 U and TRAP = 50 U), otherwise coagulation results were with a tendency to lower normal ranges.

Patient B presented only a borderline ADP value of 51 U. She also showed elevated F II (119%), F V (120%), F XII (135%), F XIII (138%) and borderline elevated FibTEM (CT = 63 mm, A5 = 127 mm) and ExTEM (A5 = 59 mm, A10 = 67 mm).

## Discussion

### Relevance of the issue

In 2021, there were 69.269 hospital admissions due to acute alcohol intoxication [18]. Entire coagulation profiles are not carried out routinely in an emergency setting. The European guidelines on management of major bleeding and coagulopathy following trauma recommend platelet count, Quick/INR, prothrombin time and fibrinogen level for screening coagulation deficits [19].

In the presented examinations comprehensive coagulation profiles were determined in persons with high BAC in order to identify possible diagnostic gaps.

### Salient findings and comparison with previous studies

In fact, alcohol-associated alterations of the platelet function, which might be clinically relevant were observed. Multiplate*®* analyses revealed a reduced aggregometric capacity especially after ADP and ASPI stimulation. Hillbom et al. [20] found that thrombocyte aggregation is only inhibited 10 min after ethanol intake and then increases again. We were unable to confirm this assumption. The blood samples were taken during treatment in the emergency room, 10 min were certainly exceeded since the beginning and also termination of alcohol intake, and our results nevertheless indicated a remaining impairment of platelet function.

ADP is a weak stimulator of platelet aggregation [21] and is altered by smallest changes in platelet function [22]. The changes seen in the ADP test during severe alcohol intoxication can be attributed to the effects of alcohol. In certain alcoholised patients, their blood showed a limited platelet function. Reliable information as to the intake of antiplatelet drugs was not available. We suppose that increasing and normalising ADP-induced aggregation was not influenced by antiplatelets as ADP-receptor antagonist medication is not frequent and the personal history of the patients under investigation was free of diseases with might have caused an indication for ADP-receptor antagonist treatment. Nevertheless, it must be considered that very low ASPI values may also be caused by ASA/NSAID analgesics [23]. Therefore, results without comparison analyses on the next day must be interpreted with caution.

Interestingly, normalisation of the aggregometric capacity during detoxication, which was observed with all types of stimulation, was complete only with ADP values (*N* = 6 each). The authors do not consider decreased aggregometric platelet capacity (to a limited extend of < 20%, with absolute values in or near to the normal range) in two of six patients pathophysiologically relevant.

In six patients with follow-up testing (see Fig. 5, 6, and 7) the drug history was complete. One patient (orange line) had taken Aspirin, which explains improved platelet function with ASPI testing, but not with TRAP or ADP. A second patient (grey line) had prior gabapentin intake, which may explain improved platelet function in all three tests in the status of soberness (after gabapentin was paused). Overall improved platelet function during detoxing seems not to be explained by decreasing drug effects; but this hypothesis has to be substantiated with a higher number of cases.

Only few studies investigated primary blood coagulation at high alcohol concentrations in-vivo [3, 5, 7]. However, most studies relate to plasmatic coagulation only.

Nguyen did not investigate the effect of ethanol on aggregation but on the adhesion of platelets to collagen using platelets isolated from human donor blood samples [24]. Ethanol was found to inhibit the formation of TXA2 and reduce collagen-induced phosphorylation of phospholipase A2. The results of our ASPI-test also indicated an influence of alcohol on the formation of TXA2. The specific influence could not be determined.

Von Willebrand factor was addressed as the second player in primary hemostasis. An increase in vWF leads to an improvement in primary hemostasis, which is therapeutically utilized in mild forms of congenital thrombopathies through the administration of desmopressin [25]. An acute-phase reaction can also lead to an increase in vWF antigen levels. However, since the other parameters (Albumin, CRP, Leukocytes) typically altered during an acute-phase reaction remained within the normal range, this explanation seems unlikely as the underlying cause. Nevertheless, if the acute-phase reaction is indeed responsible for the increase, it could suggest that the alcohol-induced mild thrombopathy was at least partially compensated in some patients by this reaction. The ratio of factor activity and antigen which reflects functionally important large multimers, was found to weakly correlate with the BAC (r = −0,41, *p* = 0.06), meaning that increasing BACs lead to lower ratios. Further investigations including multimer testing should address the potential that high BAC can impair von Willebrand factor related hemostasis.

Studies with focus on platelet function also concluded that increased ethanol consumption leads to an impairment of the primary hemostasis phase, more precisely to reduced platelet aggregation in response to ADP and collagen [26–31]. However, ethanol was sometimes consumed over several weeks and the BAC at the time of blood sampling was not as high as in the presented data set. In Smith’s study, in which the breath alcohol level was 0.08 mg/dl (approx. 1.6 g/kg), changes were only seen in men [29]. Of course, it cannot be ruled out that a higher BAC would also have resulted in a change in women. In the conducted examinations gender-related differences did not become evident. However, the data set is too small for a thorough gender-specific analysis.

When comparing the presented results of persons with trauma with those of persons without trauma, there were no remarkable differences in the various examinations. Mean ADP test values were slightly lower in persons with trauma (41.4 U) than without trauma (48.2 U), though. However, in both groups the ADP test values of alcoholised persons increased in the state of soberness. Also, the traumas were not severe enough to cause hemorrhagic coagulopathy, but they may have contributed to hemostatic activation. Overall, it remains likely that alcohol is the cause of the change of platelet function.

The authors cannot present a convincing explanation why the abnormal results of Multiplate analyses could not be provoked in the same way by ex-vitro addition of ethanol (*N* = 1). A possible explanation could be the short exposure time of alcohol on blood, so full effects could not develop. In vivo, the alcohol penetrates the human cell and is degraded there, which might be a time-depending process [32]. An interesting alternative explanation could be that the degradation product of alcohol, acetaldehyde, instead of ethanol itself leads to changes in coagulation. Follow-up studies are required to pursue these options.

As compared to light transmission aggregometry impedance aggregometry has reduced sensitivity [33]. Thus the degree of impaired platelet capacity associated with alcohol intoxication might methodologically be underestimated in our study.

An impairment of platelet function could be particularly relevant for clinicians, as precaution measures could be taken for people who come to the emergency department heavily intoxicated and with injuries to avoid being exposed to the risk of increased bleeding.

No deficits in secondary hemostasis putting alcoholised patients at a bleeding risk were seen. This is true for the results from viscoelastic testing as well as conventional testing (global tests and single factor activities).

Overall, there are indications of typical changes in coagulation at very high in vivo BACs (starting at about 2 g/l) both with and without accompanying injuries. Nevertheless, our initial question cannot be fully answered, which is partly due to the small number of subjects included. In order to gain clarification, additional research methods must certainly be considered. Concluding, there appears to be a potential trend towards an impairment of platelet function at high BAC in-vivo.

## Conclusions

- Within the limitations of the study, our findings suggest alcohol-induced changes in platelet function. Secondary hemostasis does not appear to be affected by alcohol.
- Accordingly, the focus of further studies should be on the investigation of platelet function.
- Von Willebrand factor multimer testing may clarify further impairment of primary haemostasis by high BAC
- In particular, multimeric tests should be performed to investigate whether high BAC impair the hemostasis associated with von Willebrand factor. In vitro effects of ethanol on blood coagulation are not directly comparable to the coagulation changes observed in vivo in alcoholised individuals.

## Limitations

The study is based on a comparatively small number of participants. Possible confounders of coagulation analytic (trauma – non-trauma, males—females) could not be evaluated in more detail. Many participants exhibited low adherence to medical advice. For that reason, control examinations in the state of soberness could not be realized in all cases. Results obtained without soberness control must be considered with caution, given uncertainties regarding the medication history of many participants.

## Supplementary Information

Below is the link to the electronic supplementary material.

Supplementary file1 (DOCX 22.1 KB)

## Data Availability

The datasets generated during and/or analysed during the current study are available from the corresponding author on reasonable request.
