# Supplementary material for: Evaluation of alcohol intoxication on primary and secondary haemostasis – results from comprehensive coagulation testing
Source: Int J Legal Med. 2025 Feb 22;139(4):1797–808. doi: 10.1007/s00414-025-03449-7 (PMC12170711; doi:10.1007/s00414-025-03449-7)
Supplement: Supplementary file 1 — Supplementary file1 (DOCX 22.1 KB) [file 414_2025_3449_MOESM1_ESM.docx]

| Rotem® parameter | Reference value  Numbers above  Numbers below | Average in %  patients with trauma  pat. without trauma | Median in %  patients with trauma  pat. without trauma | Range in %  patients with trauma  pat. without trauma |
| --- | --- | --- | --- | --- |
| **Fibtem** |  |  |  |  |
| CT | 28-62 sec.  2  0 | \| 54,10 \| \| --- \| \| 53,36 \| \| 54,90 \| | \| 53,00 \| \| --- \| \| 55,00 \| \| 51,50 \| | \| 31,00 \| \| --- \| \| 15,00 \| \| 30,00 \| |
| A5 | 4 - 17 mm  5  0 | \| 20,86 \| \| --- \| \| 24,27 \| \| 17,10 \| | \| 15,00 \| \| --- \| \| 15,00 \| \| 14,50 \| | \| 120,00 \| \| --- \| \| 120,00 \| \| 20,00 \| |
| A10 | 7 - 23 mm  4  0 | \| 19,29 \| \| --- \| \| 16,00 \| \| 22,90 \| | \| 16,00 \| \| --- \| \| 16,00 \| \| 16,00 \| | \| 51,00 \| \| --- \| \| 17,00 \| \| 45,00 \| |
| α | 1  0 | \| 64,89 \| \| --- \| \| 65,11 \| \| 64,70 \| | \| 70,00 \| \| --- \| \| 69,00 \| \| 70,00 \| | \| 70,00 \| \| --- \| \| 50,00 \| \| 70,00 \| |
| MCF | 9 - 25 mm  2  0 | \| 18,21 \| \| --- \| \| 17,20 \| \| 19,33 \| | \| 17,00 \| \| --- \| \| 16,50 \| \| 17,00 \| | \| 23,00 \| \| --- \| \| 14,00 \| \| 20,00 \| |
| **Extem** |  |  |  |  |
| CT | 38 - 79 sec.  1  0 | \| 55,90 \| \| --- \| \| 54,09 \| \| 57,90 \| | \| 55,00 \| \| --- \| \| 54,00 \| \| 55,50 \| | \| 43,00 \| \| --- \| \| 27,00 \| \| 37,00 \| |
| CFT | 34 - 159 sec  1  0 | \| 84,33 \| \| --- \| \| 95,18 \| \| 72,40 \| | \| 76,00 \| \| --- \| \| 80,00 \| \| 71,50 \| | \| 178,00 \| \| --- \| \| 173,00 \| \| 71,00 \| |
| A5 | 34 - 55 mm  3  2 | \| 47,43 \| \| --- \| \| 44,91 \| \| 50,20 \| | \| 50,00 \| \| --- \| \| 47,00 \| \| 50,00 \| | \| 39,00 \| \| --- \| \| 33,00 \| \| 27,00 \| |
| A10 | 43 - 65 mm  3  1 | \| 57,14 \| \| --- \| \| 54,91 \| \| 59,60 \| | \| 59,00 \| \| --- \| \| 58,00 \| \| 60,50 \| | \| 35,00 \| \| --- \| \| 30,00 \| \| 25,00 \| |
| α | 63 - 83°  0  1 | \| 74,00 \| \| --- \| \| 72,09 \| \| 76,10 \| | \| 75,00 \| \| --- \| \| 74,00 \| \| 76,00 \| | \| 31,00 \| \| --- \| \| 30,00 \| \| 14,00 \| |
| MCF | 50 - 72 mm  1  2 | \| 60,05 \| \| --- \| \| 58,18 \| \| 62,10 \| | \| 63,00 \| \| --- \| \| 61,00 \| \| 64,00 \| | \| 29,00 \| \| --- \| \| 24,00 \| \| 23,00 \| |
| **Intem** |  |  |  |  |
| CT | 100 - 240 sec  1  0 | \| 164,67 \| \| --- \| \| 167,45 \| \| 161,60 \| | \| 163,00 \| \| --- \| \| 163,00 \| \| 162,50 \| | \| 135,00 \| \| --- \| \| 113,00 \| \| 81,00 \| |
| CFT | 30 - 110 sec  0  0 | \| 73,29 \| \| --- \| \| 77,73 \| \| 68,40 \| | \| 72,00 \| \| --- \| \| 75,00 \| \| 70,00 \| | \| 66,00 \| \| --- \| \| 57,00 \| \| 66,00 \| |
| A5 | 38 - 57 mm  1  2 | \| 46,33 \| \| --- \| \| 44,55 \| \| 48,30 \| | \| 46,00 \| \| --- \| \| 44,00 \| \| 46,50 \| | \| 26,00 \| \| --- \| \| 18,00 \| \| 26,00 \| |
| A10 | 44 - 66 mm  1  0 | \| 55,81 \| \| --- \| \| 54,27 \| \| 57,50 \| | \| 56,00 \| \| --- \| \| 55,00 \| \| 56,50 \| | \| 24,00 \| \| --- \| \| 15,00 \| \| 24,00 \| |
| α | 70-83°  0  1 | \| 75,65 \| \| --- \| \| 75,10 \| \| 76,20 \| | \| 76,00 \| \| --- \| \| 75,50 \| \| 76,00 \| | \| 14,00 \| \| --- \| \| 9,00 \| \| 14,00 \| |
| MCF | 50 - 72 mm  0  3 | \| 56,80 \| \| --- \| \| 55,60 \| \| 58,00 \| | \| 57,00 \| \| --- \| \| 56,00 \| \| 57,00 \| | \| 23,00 \| \| --- \| \| 13,00 \| \| 23,00 \| |

**Supplement 1:** Results from ROTEM analyses
